# Supplementary material for: Association of remittances with skilled delivery in Uganda, 2019/2020
Source: PLOS Glob Public Health. 2025 Jun 13;5(6):e0004769. doi: 10.1371/journal.pgph.0004769 (PMC12165348; doi:10.1371/journal.pgph.0004769)
Supplement: S1 Table — (DOCX) [file pgph.0004769.s001.docx]

**Supporting information**

**S1 Table. Description of study measures: outcome, exposure, and covariates**

| **Measure** | **Section** | **Variable Type** | **UNPS Question** | **Survey Responses (taken directly from Uganda National Panel Survey)** |
| --- | --- | --- | --- | --- |
| *Exposure - Remittances* | | | | |
| Receipt of remittances | Household Questionnaire | Categorical | What is the household’s **most** important source of earnings during last 12 months? | 1= Subsistence farming 2= Commercial farming 3= Wage employment 4= Non-agricultural enterprises 5= Property income 6= Transfers (pension, allowances, social security benefits,) 7= Remittances 8= Organizational support (e.g., food aid, WFP, NGOs etc.) 9=SAGE 96=Other (specify) |
|  | ^&^Household Questionnaire | Binary | Has the household received any income (in cash &in kind) from […] in the past 12 month–? - Remittances and assistance received locally (elsewhere in the country) | 1= Yes 2= No |
|  | ^&^Household Questionnaire | Binary | Has the household received any income (in cash &in kind) from [...] in the past 12 months? - Remittances and assistance received from abroad | 1= Yes 2= No |
|  | Household Questionnaire | Continuous | Amount received during the past 12 months. If amount was in kind, give the estimated cash value. | Numeric Value |
| *Proportion of Household Income | Household Questionnaire | Continuous | Total amount remittance/Total Household Consumption | Numeric Value (calculated) |
| *Outcomes – Maternal Healthcare Utilization, Child Immunization, Vitamin A, Child Stunting and Wasting* | | | | |
| Skilled Delivery | Woman Questionnaire | Categorical | Who assisted with the delivery of (NAME)? | HEALTH PERSONNEL Doctor….............................................A  Nurse/Midwife....................................B  Medical Assistant/ Clinical Officer......C  Nursing Aide ......................................D  OTHER PERSON Traditional Birth Attendant..................E  Relative/Friend ...................................F  NO ONE .............................................X  OTHER (SPECIFY).............................Y |
|  | Woman Questionnaire | Categorical | Were you provided with a MAMA Kit? | 1=Yes, free of charge 2=Yes, at a fee 3=No 98=DK |
| ^&^Location of delivery | Woman Questionnaire | Categorical | Where did you give birth to (NAME OF LAST CHILD)? | HOME 1 = Your Home 2 = TBA's Home 3 = Other Home PUBLIC SECTOR 4 = Govt. Hospital 5 = Govt. Health Center 6 = Govt. Health Post PRIVATE MED. SECTOR 8 = Pvt. Hospital/Clinic 86 = Other Private Med, (Specify) 96 = Other Public, (Specify) 76 = Not Sure, (Specify name of hospital, clinic, etc.) |
| Child Anthropometry | Household Questionnaire | Continuous | *WEIGHT OF HOUSEHOLD MEMBER* | Numeric Value (Kg) |
|  | Household Questionnaire | Continuous | *LENGTH (CM) LYING DOWN/ HEIGHT (CM) STANDING UP* | Numeric Value |
|  | Household Questionnaire | Continuous | Stunting (calculated with height and age) | Numeric Value |
|  | ^&^Household Questionnaire | Continuous | Wasting (calculated with weight and height) | Numeric Value |
| Child Illness | Household Questionnaire | Binary | During the past 30 days, did [NAME] suffer from any illness or injury? | Yes = 1 No = 2 |
|  | Household Questionnaire | Categorical | Can you describe the symptoms that [NAME] primarily suffered due to the major illness or injury during the past 30 days? | RECORD UP TO 2 SYMPTOM CODES  1= Diarrhoea (acute) 2= Diarrhoea (chronic, 1 month or more) 3= Weight loss (major) 4= Fever (acute) 5= Fever (recurring) 6= Wound 7= Skin rash 8= Weakness 9= Severe headache 10= Fainting 11= Chills (feeling hot and cold) 12= Vomiting 13= Cough 14= Productive cough 15= Coughing blood 16= Pain on passing urine 17= Genital sores 18= Mental disorder 19= Abdominal pain 20= Sore throat 21= Difficulty breathing 22= Burn 23= Fracture 40= Nothing else 96= Other (specify) |
|  | Household Questionnaire | Binary | Was anyone consulted (e.g., a doctor, nurse, pharmacist or traditional healer) for the major illness/ injury [NAME] suffered during the past 30 days? | Yes = 1 No = 2 |
|  | Household Questionnaire | Categorical | Why was no one consulted for the major illness? | 1= Illness mild 2= Facility too far 3= Hard to get to facility 4= Too dangerous to go 5= Available facilities are too costly 6= No qualified staff present 7= Staff attitude not good 8= Too busy / long waiting time 9= Facility is inaccessible 10= Facility is closed 11= Facility is destroyed 12= Drugs not available 96= Other (specify) |
| *Covariates* |  |  |  |  |
| Migration | Household Questionnaire | Categorical | If [NAME] has not stayed for 12 months, what is the main reason for absence? | **New arrivals** 1=New born 2=Returned home from abduction/displacement 3=To escape insecurity from home area 4=Bad living conditions at home 5=To look for work 6=Other economic reasons 7=Education 8=Marriage 9=Divorce **Members that left** 10=Deceased 11=To escape insecurity from this area 12=Looking for work elsewhere 13=Other economic reasons 14=Illness 15=Education 16=Marriage 17=Divorce 18=Started own household 19=Abducted/disappeared 20=Other (specify) |
| Mobile Money | Household Questionnaire | Binary | Did the HH consume Mobile Money charges? | 1= Yes 2= No |
|  |  | Categorical | Which of the following transactions do you conduct for yourself normally at the Mobile money point? | A=Cash withdrawals B=Cash deposits C=Cash Transfer D=School Fees Payment E=Utility payments (Water, Power, TV) F=Purchase of airtime G=Western Union H=Mobile Banking I=Payment for goods and services J=To send money K=Receive money M=Receiving wages/salaries X=Others (Specify)  1= Yes 2= No |
| Marital Status (Household Head) | Household Questionnaire | Categorical | What is the present marital status of [NAME]? | 1= Married monogamously  2= Married polygamous  3=Divorced /Separated 4= Widow/ Widower  5= Never Married |
| # Of household members (calculated) | Household Questionnaire | Continuous | What is the residential status of [NAME]? | Count of usual and regular members  1=Usual member present 2= Usual member absent 3=Regular member present 4=Regular member absent 5=Guest 6=Usual member who left hh more than 6 months ago 7=Left permanently |
| ^&^Child Dependency Ratio (calculated by number of children in household divided by members of working age) | Household Questionnaire | Categorical | What is the relationship of [NAME] to the head of the household? | 1= Head 2= Spouse 3= Son/daughter of head or spouse  4= Grandchild 5= Parent of head or spouse 6= Sister/Brother of head or spouse  7= Nephew/Niece  8= Other relatives  10= Non-relative |
|  | Household Questionnaire | Continuous | How old is [NAME] in completed years? | Numeric Value |
| Age of Household Head | Household Questionnaire | Continuous | How old is [NAME] in completed years? | Numeric Value |
| Education of Household Head | Household Questionnaire | Categorical | What was the highest grade/class that [NAME] completed? | 10=Some schooling but not completed P.1 11=Completed P.1 12=Completed P.2 13=Completed P.3 14=Completed P.4 15=Completed P.5 16=Completed P.6 17=Completed P.7 21=Completed J.1 22=Completed J.2 23=Completed J.3 31=Completed S.1 32=Completed S.2 33=Completed S.3 34=Completed S.4 35=Completed S.5 36=Completed S.6 41=Completed Post primary specialized training or certificate 51=Completed Post secondary specialized training or diploma 61=Completed degree and above 99=Don't know |
|  | Household Questionnaire | Categorical | What grade/class is [NAME] currently attending? | 01=Attending nursery, kindergarten etc. (lower than P.1) 10=Attending P.1 11=Attending P.2 12=Attending P.3 13=Attending P.4 14=Attending P.5 15=Attending P.6 16=Attending P.7 30=Attending S.1 31=Attending S.2 32=Attending S.3 33=Attending S.4 34=Attending S.5 35=Attending S.6 40=Attending post primary/junior specialized training or certificate or diploma 50=Attending post secondary specialized training or diploma 61=Attending degree and above 99=Don't Know |
| Location (Rural/Urban) | Household Questionnaire | Categorical | Rural/Urban | 1=Urban 2=Other Urban 3=Rural |
| Gender of Household Head | Household Questionnaire | Binary | Sex | 1=M 2=F |
| Household Consumption | Household Questionnaire | Continuous | Sum of all types of consumption | Numeric Value (calculated) |
