# Supplementary material for: Association of remittances with skilled delivery in Uganda, 2019/2020
Source: PLOS Glob Public Health. 2025 Jun 13;5(6):e0004769. doi: 10.1371/journal.pgph.0004769 (PMC12165348; doi:10.1371/journal.pgph.0004769)
Supplement: Attachment — Submitted filename: Revise and resubmit_PLOS.docx [file pgph.0004769.s003.docx]

February 12, 2025

*PLOS Global Public Health*

Dear Editor and Reviewer #1,

Thank you for the opportunity to revise and resubmit our manuscript “Association of remittances with skilled delivery and child health in Uganda, 2019/2020.” Please find attached to this letter a table with responses to each of your comments. I appreciate your feedback to my manuscript and have tried to respond to your comments as clearly and thoroughly as possible.

As suggested, I made changes to the introduction to provide more clarity on the UNPS dataset. In addition, I provided justifications for the choice of births during the last 3 years, the usage of assets, further discussion on lack of household income in the analysis, and limitations of the outcomes’ dependency on data availability. In the table below, comments are listed by line number as presented in the “Review Comments to the Author.” The table includes the reviewer’s original comment, the response to the comments, and the page number of each change.

Your time to review this manuscript and your helpful comments are greatly appreciated. I hope these changes have addressed all of your concerns and made a stronger paper.

Thank you for your consideration.

Sincerely,

Authors of “Association of remittances with skilled delivery and child health in Uganda, 2019/2020”

**Table 1. Revise and Resubmit Response Table**

|  | **Reviewer’s Comments** | **Author’s Response** |
| --- | --- | --- |
| **Reviewer #1** | The introduction of this manuscript contains a few ambiguous statements and presents sentences that are more suggestions than statements. The information on the UNPS is scattered around the introduction and the source of data. Since UNPS is the only source of data, it is important to describe it in detail in the introduction or under the source of data thoroughly. | Without specific reference to ambiguous statements, I am uncertain which sentences needed the most attention. However, the introduction to the manuscript has been edited for clarity and I added transition sentences to help with flow. See lines 55-138.  Thank you for pointing out that the data source was not mentioned in the introduction. The manuscript has been edited to include information on the data source in the introduction. See lines 113-114 and 118-120. |
|  | Although remittances information for the past 12 months is used births during the last 3 years are used in the study. What would be the justification? Does this not introduce bias? | Thank you for pointing out the need for clarification. Women of reproductive age with a birth in the last 3 years were included in the analysis due to the data collection period. Households were interviewed twice between March 2019 and February 2020, with household rosters updated as needed. This means that responses to remittance questions could reference a period from March 2018 through February 2019. The goal was to capture births that could have occurred in 2018, 2019, and 2020. The inclusion criteria were kept broad in order to account for potential recall errors on month and day of birth. This does introduce potential bias, which is addressed in the limitations section of the manuscript. This information has been included in the manuscript. See lines 193-202 and 646-649. |
|  | Not being able to include the household income in the analysis is a serious draw back since assets does not always match the income. | This is true. However, the self-reported household income in the dataset was even more unreliable. There was a significant amount of missingness and for households that did report their incomes, all of the values were “too perfect.” Under those circumstances, using assets as tangible objects that individuals can identify was more reliable. See lines 638-640. |
|  | The limitations of the UNPS data set should be highlighted under the limitations more since, the outcome is totally dependent on the data available and quality of data. | Additional details regarding the limitations of the UNPS dataset have been included in the discussion of the manuscript. See lines 652-658. |
| **Line 96** | Introduction to remittances does not clearly mention what the authors refer to - local or foreign | This is a great point. I edited the text to reflect any type of monetary remittances received. See line 101. |
| **Line 110** | Objectives are presented in the present tense | The tenses have been edited to the past tense. Other sentences in this paragraph were also edited to the past tense. See lines 115-138. |
| **Line 141** | Other household variables...... it is not clear what the authors intend to say here, is this a statement or a question? | The sentence beginning in “Other household variables…” is long and had confusing structure. Thank you for noting this. The sentence has been reorganized for clarity of thought. See lines 165-167. |
| **Line 216** | These kits include items like... are these standard kits or custom made? The word like introduces ambiguity. | I agree with the ambiguity of the word “like.” I was attempting to address the variation in items included in clean birthing kits. However, I include specifics about the MAMA kits used in Uganda as directed by the Ministry of Health. See lines 247-251. |
| **Line 246** | Household and be if little use - should be corrected as "be of little use" | Thank you for pointing out this typo. The correction has been made. See line 283. |
